# Hyperactivation of the proteasome in *Caenorhabditis elegans* protects against proteotoxic stress and extends lifespan

**DOI:** 10.1016/j.jbc.2022.102415

**Published:** 2022-08-23

**Authors:** Raymond T. Anderson, Thomas A. Bradley, David M. Smith

**Affiliations:** Department of Biochemistry, West Virginia University School of Medicine, Morgantown, West Virginia, USA

**Keywords:** Caenorhabditis elegans, enzyme kinetics, oxidative stress, proteotoxic stress, proteasome, protein degradation, ubiquitin, toxicity, aging, AMC, 7-amino-4-methylcoumarin, FP, fluorescence polarization, HS, heat shock, MW, molecular weight, ND, neurodegenerative disease, UPS, ubiquitin proteasome system

## Abstract

Virtually all age-related neurodegenerative diseases (NDs) can be characterized by the accumulation of proteins inside and outside the cell that are thought to significantly contribute to disease pathogenesis. One of the cell’s primary systems for the degradation of misfolded/damaged proteins is the ubiquitin proteasome system (UPS), and its impairment is implicated in essentially all NDs. Thus, upregulating this system to combat NDs has garnered a great deal of interest in recent years. Various animal models have focused on stimulating 26S activity and increasing 20S proteasome levels, but thus far, none have targeted intrinsic activation of the 20S proteasome itself. Therefore, we constructed an animal model that endogenously expresses a hyperactive, open gate proteasome in *Caenorhabditis elegans*. The gate-destabilizing mutation that we introduced into the nematode germline yielded a viable nematode population with enhanced proteasomal activity, including peptide, unstructured protein, and ubiquitin-dependent degradation activities. We determined these nematodes showed a significantly increased lifespan and substantial resistance to oxidative and proteotoxic stress but a significant decrease in fecundity. Our results show that introducing a constitutively active proteasome into a multicellular organism is feasible and suggests targeting the proteasome gating mechanism as a valid approach for future age-related disease research efforts in mammals.

Aging is a biological process that happens in all multicellular organisms over time and is characterized by the chronological accumulation of cellular damage leading to functional decline as an organism grows older. Functional decline may be caused by many coinciding systemic, cellular, and molecular factors, but one specific inevitability that afflicts humans as we age is the disruption of cellular proteostasis leading to the buildup of damaged and aggregate prone proteins (1, 2, 3). The buildup of such non-native proteins is an important hallmark of aging thought to contribute to organismal decline and has been linked to several age-related diseases (1, 3, 4, 5). Two systems exist in the cell that are responsible for degrading misfolded and damaged proteins: the ubiquitin proteasome system (UPS) and the lysosome (autophagy). The UPS is highly regulated and is responsible for degrading individual misfolded, damaged, or unneeded proteins, while the lysosome is responsible for degrading larger cargo including organelles and large protein aggregates. Many age-related diseases, including virtually all neurodegenerative diseases (NDs), can be characterized by protein misfolding and accumulation, and in many cases, these diseases have also been shown to have decreased UPS and autophagic functions (6, 7, 8, 9, 10, 11, 12, 13). The definitive cause of decreased proteasome function has yet to be determined. However, proteasome activity has been shown to decrease with age, which could lead to protein accumulation, especially later in life when NDs primarily occur; a wide range of literature supports this hypothesis (14, 15, 16, 17, 18, 19, 20, 21, 22, 23). In agreement with this notion, synthetic proteasome impairment alone in mice and rats has been shown to cause pathologies and symptoms associated with NDs (24, 25, 26, 27, 28). To better understand the potential mechanisms of proteasome impairment in disease, understanding how the proteasome regulates substrate degradation is needed.

The 20S proteasome, the proteolytic component, is a barrel-like structure with four stacked heptameric rings (⍺_7_, β_7_, β_7_, ⍺_7_) (29). The ⍺-rings’ N termini form “gates” that deter nonspecific degradation by interacting with one another to form a folded structure over the central pore preventing unregulated substrate entry (30). The gate’s structural stability is mediated by the evolutionarily conserved YDR motif (Tyr-Asp-Arg[Ser]), which stabilizes both the closed and open states of the 20S substrate gate (29, 30, 31, 32, 33). The β-rings house two copies of three distinct proteolytic sites that cleave after: hydrophobic (chymotrypsin-like), basic (trypsin-like), and acidic (caspase-like) residues. Several different regulatory caps that bind to the 20S proteasome exist in the cell to aid in regulating protein degradation by the proteasome (*i.e.*, PA200/Blm10, PA28αβ, PA28γ, 19S), and many of these caps have been extensively characterized (34, 35, 36, 37, 38). The 19S regulatory particle is one of the primary regulatory caps in the cytosol. It binds to one or both ends of the 20S proteasome creating the 26S proteasome. The 19S consists of a base and lid region. The base consists of a hexameric ring of AAA-ATPases for protein unfolding, and the lid contains ubiquitin receptors and deubiquitinases for targeting substrates for degradation (39, 40, 41, 42). The C termini of the ATPase subunits contain a HbYX (hydrophobic, tyrosine, most amino acids) motif, which docks into intersubunit pockets in the 20S complex causing conformational changes in the 20S α-subunits triggering gate opening (43).

Many previous studies have reported that one cause of proteasomal inhibition in aging and disease states is the presence of oligomeric proteins and other types of protein aggregates (10, 44, 45, 46, 47, 48). Our lab has recently shown that conformationally specific oligomeric forms of misfolded ND-associated proteins (*i.e.*, amyloid-β, α-synuclein, and huntingtin) can bind to and inhibit 20S proteasome activity by stabilizing a closed gate conformation even in the presence of the gate opening HbYX motif (49). However, this inhibition can be reversed with saturating levels of HbYX motif peptides, highlighting the potential therapeutic opportunity in targeting the proteasomal gating mechanism (49).

Recent studies have targeted proteasome activation or upregulation as a type of therapy to combat ND or increase resistance to cellular stress (50, 51, 52, 53, 54). In fact, recent findings have shown that some FDA approved drugs can alter posttranslation modifications on the 26S proteasome that modulate its activity (52). Other studies have shown that overexpression of the 20S β5 subunit increases total proteasome levels resulting in an increase in lifespan and resistance to cellular and organismal stressors (55, 56, 57). While this demonstrates the protective effects of increasing proteasome amounts, we sought to stimulate the intrinsic activity of endogenous 20S proteasomes. A previous study in yeast has shown that an 11 residue truncation of the ⍺3’s N terminus creates a 20S with a constitutively open proteasomal gate, which leads to dramatically increased 20S peptide hydrolysis (30). More recently, it was shown that the expression of this proteasome construct in the mammalian HEK293 cell line leads to resistance to proteotoxic stress induced by tau overexpression (58). It is important to note that the truncation of α3 in HEK293 cells was exogenously overexpressed on a WT α3 subunit background (58). It was reported that the modified α3 incorporated into the 20S proteasome well, but there still may be a small population of WT proteasomes present. Thus far, no animal model has ever been made with a similar gate opening mutation. Creating this mutant in a multicellular organism poses many potential issues, as regulated protein degradation by the proteasome is imperative for almost every cellular process including immune response, signal transduction, development, metabolism, and progression through the cell cycle (59, 60, 61). To this point, Bajorek *et al.* showed that expression of this open gate proteasome in yeast hindered exit from stationary phase thereby reducing population growth following nutrient deprivation. However, in the logarithmic phase, where nutrients are readily available, population growth and cell division appear normal (62). In the present study, we generated the very first multicellular organism, *Caenorhabditis elegans*, that expresses an endogenous open gate proteasome through direct genome editing. We examine how this hyperactive proteasome affects *C. elegans* biology and impacts its lifespan and resistance to oxidative and proteotoxic stresses.

## Results and discussion

### Mutation design

**Figure 1:**
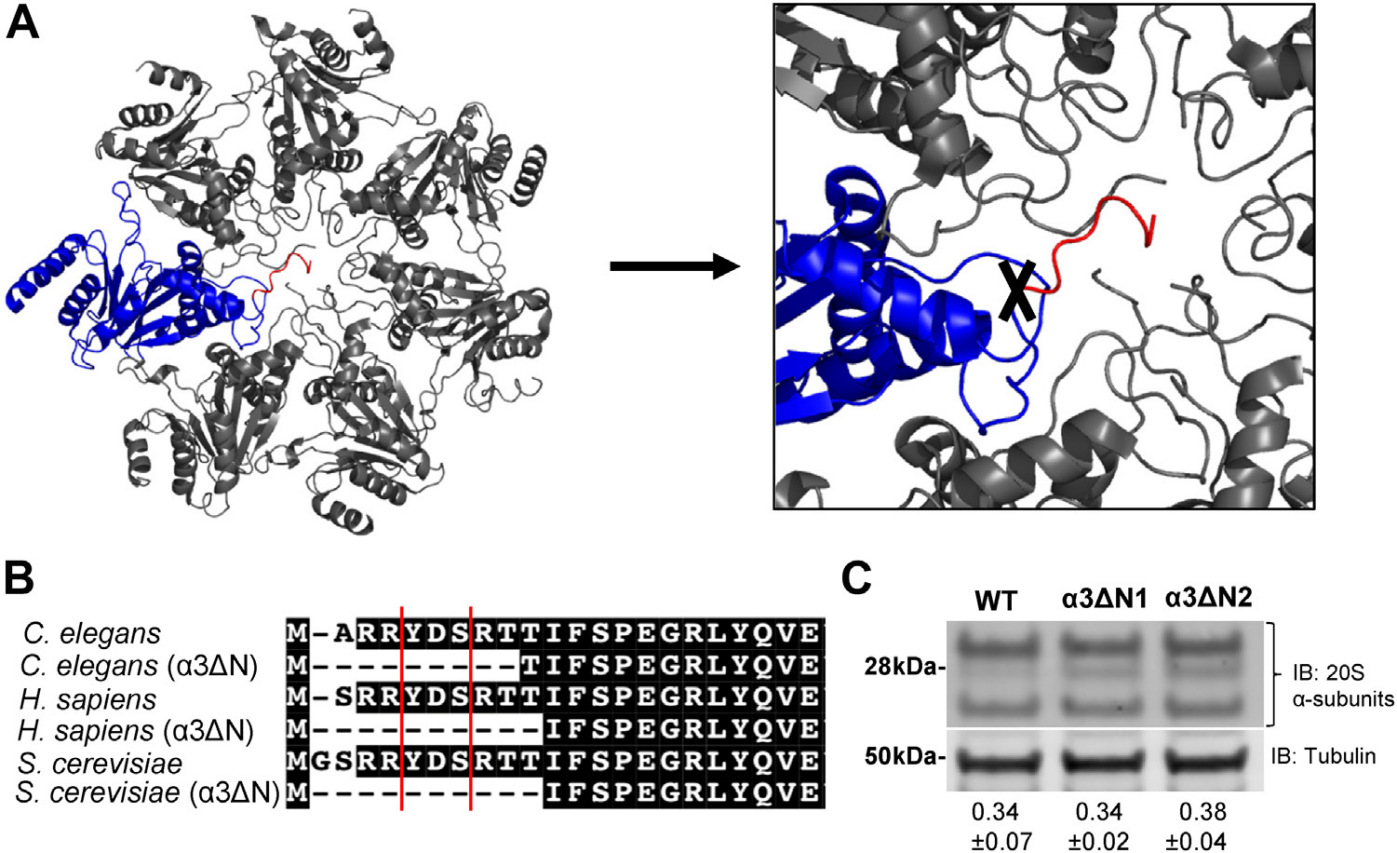
**Generation of *C. elegans* with open gate 20S proteasome.***A*, top view of *S. cerevisiae* 20S (1RYP) α-ring with α3 subunit in *blue* and its N-terminal gating residues that were removed by gene editing illustrated in *red* and cut site marked with a *black* “X”. *B*, 20S α3 N-terminal sequence alignment of *C. elegans*, *H. sapiens*, and *S. cerevisiae* showing homology, the YDR/S motif demarcated between the *red lines*. Specific residues deleted to form an open gate α3ΔN-20S in each species is indicated by the *dashed line* (30, 58). *C*, SDS-PAGE Western blot probing for 20S α-subunits (antiproteasome α's1,2,3,5,6,7; MCP231) using α-tubulin (anti-Tubulin YOL1/34, ab6161) as a loading control (N = 3, *p* < 0.05). Values given are the average relative proteasome abundance in each strain relative to tubulin ± SD.

The seven α-subunits of the eukaryotic proteasome contain N-terminal regions that fold over the central pore closing it off to prevent unregulated substrate entry. The N-terminus of each α-subunit, while highly conserved, differs slightly in sequence, length, and structure, and therefore, plays a unique role in regulating gate closure. The N-terminus of α3 is uniquely important for gating in that it extends across the length of the entry pore acting as an anchor by providing hydrogen bonding between the other α-subunit N-termini resulting in a stable closed gate conformation (29) (Fig. 1*A*). Given the high sequence conservation of the α3 N-terminus among eukaryotes (Fig. 1*B*), we hypothesized that making this mutation in nematodes would also induce gate opening and stimulate proteasome activity as has been shown in yeast and mammalian cells (30, 58). Using Co-CRISPR Cas9 technology with the assistance of InVivo Biosystems, we were able to directly edit the genome of *C. elegans* to generate a nematode population with endogenous expression of a hyperactive, open gate proteasome.

Two identical mutant clones were generated separately to control for off-target editing resulting in the strains COP1857 *pas-3(knu746 [NTD del])* and COP1858 *pas-3(knu747 [NTD del])*, which we will refer to as α3ΔN throughout this study. In these mutants, 36 base pairs were deleted and replaced with 12 base pairs of new coding (for PCR Genotyping) resulting in an 8 residue N-terminal deletion of the α3 subunit including the YDR motif (Fig. S1, *A* and *B*). Whole genome sequencing on both strains confirmed a successful α3 N-terminal truncation, and a bioinformatic analysis confirmed no editing in off-target regions for both clones (Fig. S1*C*). As determined by Western blot, native-PAGE showed proper assembly of the α3ΔN 20S (Fig. S2*A*), and SDS-PAGE showed no detectible difference in expression compared to WT (Figs. 1*C* and S2, *B* and *C*). Unchanged proteasome levels are important to note as any differences in proteasome activity detected is not due to major changes in expression but to the activity of the proteasome itself.

### Proteasome activity

**Figure 2:**
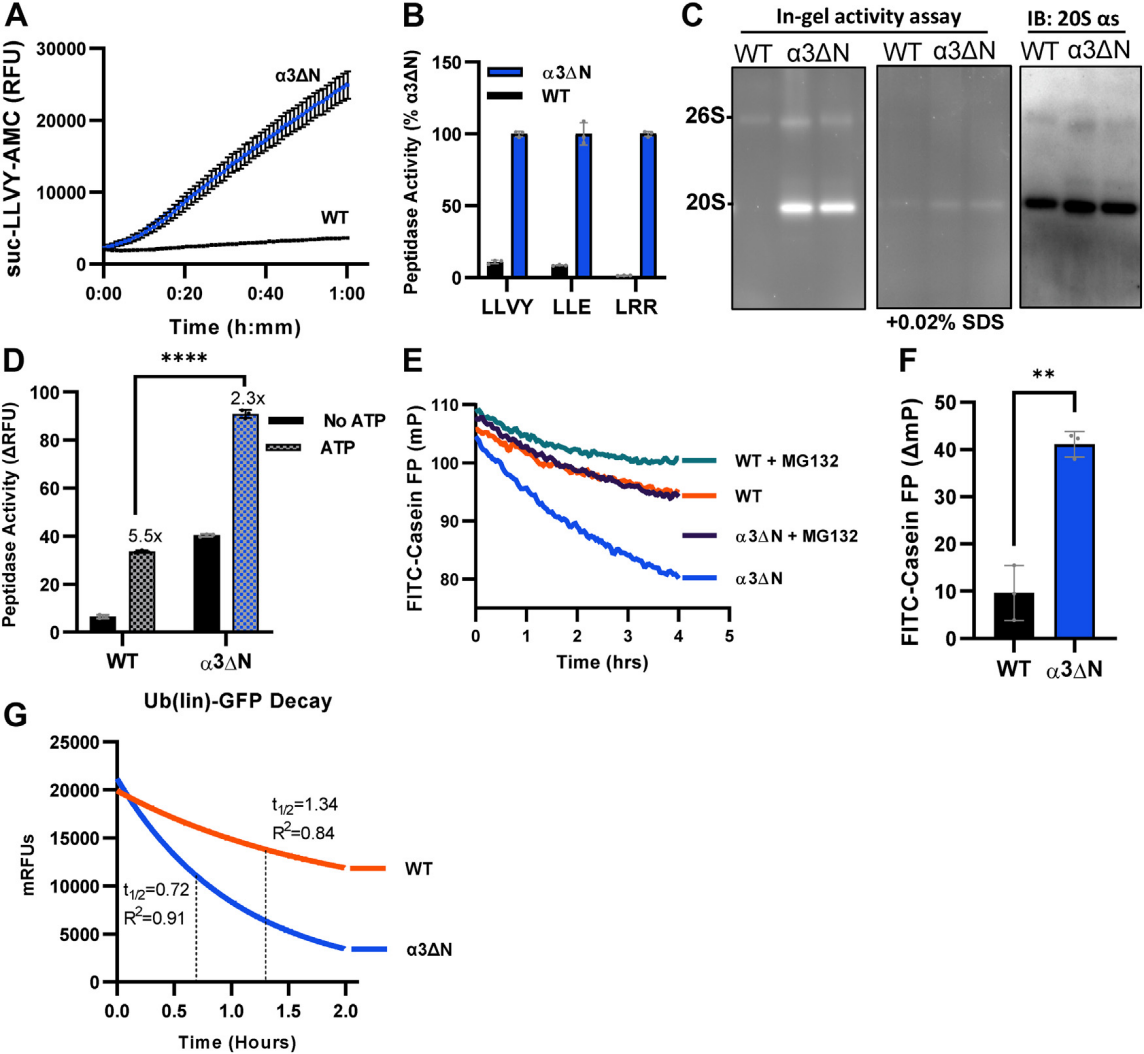
**The nematode α3ΔN proteasome is hyperactivated *in vitro*.** All activity assays were performed using nematode lysates from synchronized young adult populations. All quantitative data shown are normalized to MG132 inhibition. *A*, raw kinetic data of Suc-LLVY-AMC hydrolysis by lysates prepared using 20S lysis buffer. Fluorescence (ex/em: 380/460 nm) was measured every 60 s for 2 h (1 h shown). Values shown are average relative fluorescence units (RFUs) at each time point ± SD normalized to MG132 inhibition (N = 3). *B*, quantified peptidase activity using fluorogenic substrates targeted to all three proteasomal active sites: chymotrypsin-like (Suc-LLVY-AMC), caspase-like (ac-LLE-AMC), trypsin-like (Boc-LRR-AMC). Data are shown as percentage of mean α3ΔN activity ± SD normalized to MG132 inhibition (N = 3). *C*, native-PAGE (4%–8% Tris-acetate gel) of lysates from day 1 adults showing in-gel peptidase activity after incubation with 50 μM Suc-LLVY-AMC in 26S activity buffer (includes ATP) (*left*), peptidase activity after incubation with 0.02% SDS to induce gate opening (*middle*) and immunoblot for 20S α-subunits (antiproteasome α's1,2,3,5,6,7; MCP231) (*right*). Experiments were performed in triplicate and images shown include 1 replicate of each strain (WT, α3ΔN1, α3ΔN2). *Arrows* indicate suspected PA200-20S band. *D*, quantified peptidase activity (Suc-LLVY-AMC hydrolysis, rfu/min) using lysates with and without ATP. Values shown are average degradation rate ± SD normalized to MG132 inhibition (N = 3). Unnormalized data shown in Fig. S4. *E*, fluorescence polarization (FP) of FITC-casein over 4 h using 5 μg lysate from each strain in the presence of ATP (100 μM MG132 used to inhibit proteasome activity). Values are the second order smoothing of the raw FP at each time point (N = 3). *F*, total change in FP (ΔmP) of FITC-casein after 4 h. Values represent the average ΔmP ± SD after normalizing to MG132 inhibition (N = 3). *G*, exponential decay curve of ub_4_(lin)-GFP-35 degradation with half-life (t_1/2_) and R^2^ value labeled for each curve, normalized to MG132 inhibited. Unnormalized data shown in Fig. S4. (N = 3). All experiments were performed in triplicate at least twice and error bars represent ±SD; ∗∗*p* ≤ 0.01, ∗∗∗*p* ≤ 0.001, ∗∗∗∗*p* ≤ 0.0001. AMC, 7-amino-4-methylcoumarin.

After confirming the successful α3 N-terminal truncation and unchanged expression levels, we sought to determine whether this mutation did, in fact, cause increased proteasome activity in α3ΔN compared to WT. 20S activity can be measured *in vitro* using small fluorogenic peptides conjugated to 7-amino-4-methylcoumarin (AMC), which becomes fluorescent following cleavage from specific 3 to 4 amino acid peptides. Therefore, activity can be measured by determining the rate of increased fluorescence over time. Gate opening, rather than active site activation, can be validated by determining the stimulation of activity at all three of the 20S’s β catalytic sites using Suc-LLVY-AMC (chymotrypsin-like), Z-LLE-AMC (caspase-like), and Boc-LRR-AMC (trypsin-like). Using the highly proteasome specific substrate, Suc-LLVY-AMC, and the proteasome inhibitor, MG132, to normalize to proteasome-specific proteolysis, we observed a 13-fold increase in 20S activity in α3ΔN lysates compared to WT (Fig. 2*A*). Z-LLE-AMC and Boc-LRR-AMC were also hydrolyzed faster by α3ΔN as expected for an open gate proteasome (Fig. 2, *A* and *B*). In fact, α3ΔN lysates were as much as 50Xs more active than WT (WT was 98% less active for Boc-LRR-AMC), indicating extensive increase in proteasomal peptidase activity. Moreover, this result demonstrates that all three of these peptides are relatively specific for the proteasome in *C. elegans* lysates, since the 20S mutation substantially increased peptide hydrolysis as expected, and the activity reported was sensitive to the proteasome inhibitor MG132.

To directly confirm the increased activity was due to proteasome activation specifically, we performed an in-gel activity assay that shows proteasome peptidase activity after separating the lysate *via* native-PAGE. Both α3ΔN clones showed drastically higher 20S signal compared to WT, which was hardly detectable (Fig. 2*C*). The 26S peptide hydrolysis activity was also elevated in the α3ΔN clones relative to WT after normalizing to 26S protein levels observed *via* immunoblot (Fig. 2*C*). After the addition of 0.02% SDS, which induces gate opening in the WT 20S (43, 63, 64), we observed an equalization of the signal between WT and α3ΔN 20S, further implicating gate opening as the primary means of activation in these mutants (Figs. 2*C* and S3). We also observed a light band above the 20S likely corresponding to a PA200-20S complex, a proteasomal activator that has been reported to bind more readily to open gate proteasomes (65).

To determine the extent to which α3ΔN in *C. elegans* opens the 20S gate, we examined the activation capacity of the 26S proteasome. Typically, the 26S proteasome can be activated by 4- to 5-fold in the presence of ATP (31), which induces gate opening. When we added ATP to WT or α3ΔN lysates, we found that it stimulated Suc-LLVY-AMC peptide substrate entry by 5.5-fold for WT and 2.3-fold for α3ΔN lysates, suggesting that the mutation may not cause completely stable gate opening in these proteasomes since the α3ΔN 26S can become more active when saturated with ATP (Figs. 2*D* and S4). In agreement, prior studies using the mammalian open gate 20S also show the 19S bound α3ΔN can reach a higher level of “full” activation compared to 19S bound to WT 20S (58). The observation that mutant lysates can be further stimulated by ATP suggests that α3ΔN proteasomes may still offer a level of regulation that may contribute to the viability of these mutant populations. It is possible that a completely open gate would be lethal in a multicellular organism given the crucial role of proteasomal regulation during development (see later for more). Another open gate mutant has been generated in yeast with a truncation in both the α3 and α7 N termini (α3α7ΔN), resulting in more extensive activation than α3 truncation alone (62). We attempted to introduce this construct in *C. elegans* and were only able to produce heterozygous populations, suggesting homozygosity is lethal. This observation is consistent with the hypothesis that our α3ΔN 20S mutant is not fully open, which may contribute to the successful generation of this organism.

Thus far, we have shown 20S activity *in vitro* using only small fluorogenic peptides, which is useful because it provides information about degradative capacity and the degree to which α3ΔN elicits gate opening. However, it does not necessarily provide direct evidence α3ΔN 20S’s ability to degrade more physiologically relevant substrates such as intrinsically disordered proteins (IDPs) and polyubiquitinated proteins. Protein degradation increases the mobility of fluorescently labeled peptides that can be measured with fluorescence polarization (FP) by assessing the rotational rates of the fluorophore. We used FP to monitor degradation of the of the unstructured FITC-casein. With ATP, we observed a 4-fold higher change in FP in α3ΔN compared to that of WT (Fig. 2, *E* and *F*), demonstrating an increased capacity of α3ΔN lysates to degrade IDPs (*i.e.*, casein) under physiologic conditions. When the identical experiment was repeated without ATP, FITC-casein degradation was undetectable in WT, while degradation was clearly visible in α3ΔN (Fig. S4*B*), demonstrating that the free α3ΔN 20S (ATP-independent activity) has increased IDP degradative capacity as expected. While enhanced degradation of unfolded substrates is expected with a more open proteasome gate, it is not expected that α3ΔN proteasome would stimulate degradation of folded substrates since folded domains must be unfolded by 19S ATPases before entering the 20S, which is known to be rate limiting. To assess ubiquitin-dependent degradation capacity, we used ub_4_(lin)-GFP-35, which contains an N-terminal linear tetraubiquitin chain fused to a circularly permuted GFP with a 35 residue unstructured initiation site on its C-terminus (42). Surprisingly, the α3ΔN lysates unfolded and degraded this substrate nearly twice as fast as the WT lysates (Figs. 2*G* and S4*C*). In agreement with these findings, open-gate 20S expression in mammalian cells (in a WT proteasome background) also showed enhanced ubiquitin-dependent degradation of a protein substrate (58). These combined findings in two very different systems provide compelling evidence that gate opening can enhance ubiquitin-dependent protein degradation. While the mechanism behind this observation is not understood, perhaps enhanced substrate entry into the 20S during initial unfolding events contributes, as previously suggested (58).

### Lifespan and other notable phenotypes

**Figure 3:**
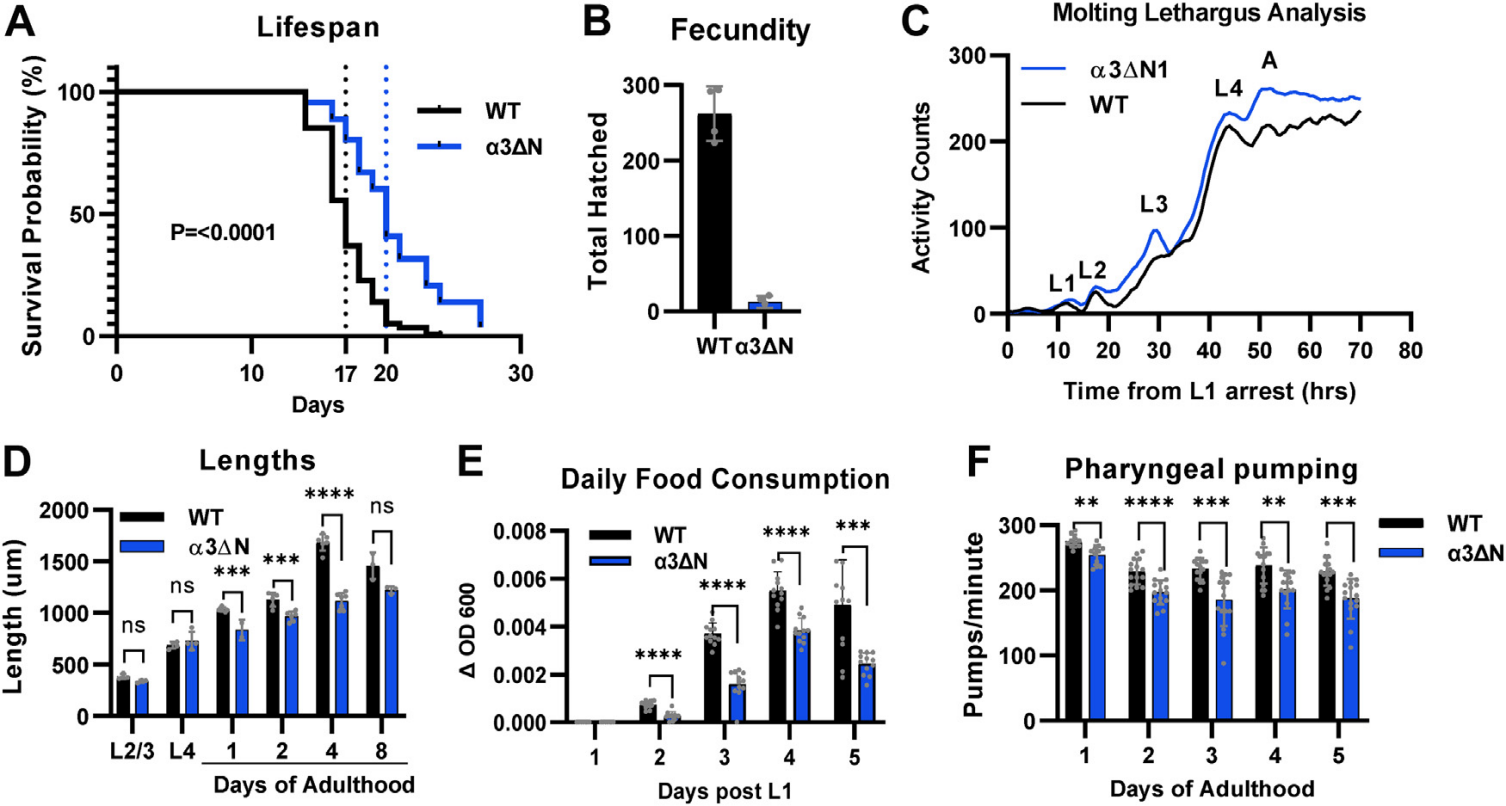
**Lifespan extension in α3ΔN and other notable phenotypes.***A*, Kaplan–Meier curve of survival for α3ΔN (N = 139) and WT (N = 121) (logrank test, *p* < 0.0001). *B*, average number of hatched progenies for each strain (N = 4, *p* < 0.0001). *C*, activity counts from wMicroTracker throughout larval development showing lethargus and peak activity for each larval stage. Data represent a second order smoothing of activity counts over 72 h (N = 12). *D*, average length measurements throughout development to day 8 of adulthood (N ≥ 3) ± SD. *E*, daily consumption of OP50 *E. coli* (Δ*A*_600_) following L1 arrest ± SD (N = 12). *F*, pharyngeal pumping (pumps/min) during the first 5 days of adulthood (N = 15). The data are representative of two or more independent experiments. Error bars represent ±SD; ∗∗*p* ≤ 0.01, ∗∗∗*p* ≤ 0.001, ∗∗∗∗*p* ≤ 0.0001.

After confirming the viability and proteasome activation in α3ΔN, we sought to characterize their phenotypic differences relative to WT. As mentioned previously, a wide range of literature reports that proteasome activity declines with age in many model systems (14, 15, 16, 17, 18, 19, 20, 21, 22, 23), and its inhibition leads to a dramatically decreased lifespan in *C. elegans* (66). In addition, many long-lived nematode mutants are characterized by increased proteostasis (67), which has been linked in some (*i.e.*, *dnj-21* and *glp-1*) to increased UPS activity (68, 69). Thus, we asked if hyperactivation of the proteasome core particle could impact lifespan. Visualized using a Kaplan–Meier curve, we found that open gate mutants have a median lifespan of 20 days compared to 17 days for WT nematodes corresponding to a 20% lifespan extension for α3ΔN (Fig. 3*A*, *p* < 0.0001). This is consistent with previous studies where proteasome upregulation increases cellular viability in mammalian systems (70) and extends the lifespans of *C. elegans* and *Drosophila melanogaster* (56, 57). However, the mechanisms of activation studied in the context of *C. elegans* and *D. melanogaster* lifespan extensions have relied on β5 subunit overexpression to increase total proteasome levels, not activation of the proteasome itself. The data shown here confirms that intrinsic proteasome activation *via* gate opening can also extend lifespan.

While open gate proteasome expression resulted in no obvious physical abnormalities and an increased lifespan, we did notice a slower population growth in α3ΔN. To investigate this, we calculated the average number of viable offspring per nematode and found a >90% decrease in fecundity for α3ΔN compared to WT (Fig. 3*B*). This contrasts with a previous study showing that increased proteasome levels (20S and 26S) in *C. elegans* elicited a 12% increase in fecundity (56). This dichotomy demonstrates that upregulation of proteasome amounts with fully intact gating residues is not physiologically analogous to proteasome activation *via* gate opening. Clearly, the loss of the ability to close the proteasome gate has a negative impact on reproductive and/or developmental systems; however, the disruptions are limited as some embryos do survive to adulthood. This decrease in fecundity could potentially contribute to the increased lifespan seen in α3ΔN as fertility and lifespan are typically inversely related (71).

Slower development is also associated with increased lifespan in many strains (72) so we sought to determine if α3ΔN’s developmental timeline was delayed. *C. elegans* pass through four larval stages (L1-L4) before reaching adulthood, each of which consisting of a “lethargus” period, where feeding and locomotion are transiently arrested during the molting process (73). Using a specially designed nematode wMicrotracker (InVivo Biosystems) to detect nematode activity levels in liquid culture, we found no significant developmental timeline differences between α3ΔN and WT with both strains going through their developmental “lethargus” periods synchronously and reaching adulthood within 50 h (Fig. 3*C*). In addition to identical developmental progression, we also found that the two strains remained the same length to each other and grew at the same rate through each developmental stage measured, which further supports the consistent developmental timeline. However, after reaching adulthood and throughout the gravid period, α3ΔN remained consistently shorter in length than WT (Fig. 3*D*). The precise reason for this in unclear but could be attributed to fewer eggs present in α3ΔN as described previously or a decreased cell size due to differences in overall food consumption. We calculated *Escherichia coli* (OP50) consumption rates by measuring daily changes in *A*_600_ and found that α3ΔN consumed less than WT (Fig. 3*E*). We then measured pharyngeal pumping (pumps/min) to determine if this correlated with α3ΔN’s decrease in overall food consumption and found that α3ΔN had consistently lower pumping frequency compared to WT over the first 5 days of adulthood (Fig. 3*F*). It is tempting to draw connections between reduced caloric intake (potentially due to increased satiation or pharyngeal pumping defects) and increased lifespan, as this is a constant theme in other studies and model systems (74), including humans. For example, mutations in *eat-2* cause decreased food consumption by directly decreasing pharyngeal pumping rates and thus food consumption that leads to a significant increase in lifespan (75, 76). However, the pharyngeal pumping rates are ∼90% lower in the *eat-2* mutants (76) compared to WT, while the α3ΔN pumping rates are only ∼10% lower than WT (Fig. 3*F*). Therefore, it is unlikely that the slight decrease in pharyngeal pumping rates in the α3ΔN is causing a caloric restriction phenotype. Nonetheless, the data presented here demonstrate that expression of an open gate proteasome in *C. elegans* is viable, increases lifespan, and the only “major” physiological deficiency found is decreased fecundity as the other phenotypic differences shown here are relatively small, even though statistically significant.

### Resistance to paraquat

**Figure 4:**
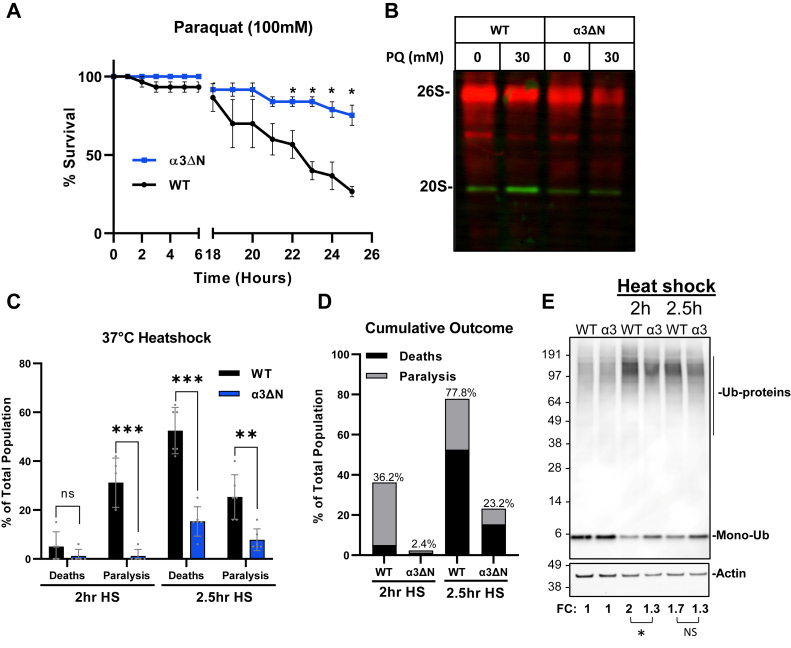
**α3ΔN are resistant to paraquat and heat shock (HS).***A*, synchronized L4 nematodes exposed to 100 mM paraquat on solid agar for 25 h with survival counted every hour (N = 5). Values are mean survival ± SEM (N = 5). The data shown are representative of two independent experiments. *B*, native-PAGE immunoblot for 20S (antiproteasome α's1,2,3,5,6,7; MCP231) and 26S (anti-PSMD7/Mov34, ab140428) complexes with and without indicated paraquat treatment. The image shown is representative of two independent experiments. *C*, young adult nematodes scored as dead or paralyzed 17 h after a 2 h and 2.5 h 37 °C HS. Bar graphs represent the average amount dead or paralyzed between five plates (20 nematodes each) ± SD; ∗∗*p* ≤ 0.01, ∗∗∗*p* ≤ 0.001, ∗∗∗∗*p* ≤ 0.0001. *D*, population (%) dead or paralyzed under each time point to visualize the cumulative outcome. *E*, SDS-PAGE immunoblot showing polyubiquitin accumulation (antiubiquitin Ubi-1, ab7254) after HS with actin (anti-βActin, AC026) as a loading control. Fold control (FC) quantifies the ubiquitinated proteins based on the respective control lane (N = 2). Statistical significance was calculated using two-way ANOVA and *p* ≤ 0.05 was considered statistically significant. Image shown is representative of two biological replicates. Additional details shown in Fig. S5. ∗*p* ≤ 0.05.

Paraquat is a potent herbicide, which produces reactive oxygen species in eukaryotes through mitochondrial disruption (77) and has been used in studies with eukaryotes including *C. elegans* as a toxin induced Parkinson’s disease model (78, 79, 80). In the context of proteostasis, oxidative stress has been observed to cause the dissociation of 19S from the 20S (54), and several studies have shown that both the 20S (54, 81, 82, 83, 84) and 26S (85, 86) are responsible for the degradation of oxidatively damaged proteins. With this in mind, we sought to determine whether our open gate strain displayed resistance to paraquat. We exposed our nematode population to 100 mM paraquat on solid NGM agar and found that after 25 h, 75% of the α3ΔN population survived, whereas only 30% of the WT population survived (Fig. 4*A*). These results clearly demonstrate that this gate opening mutation in *C. elegans* provides protection from the oxidative toxin. In addition, a native-PAGE immunoblot for 20S and 26S after 20 mM paraquat treatment showed that paraquat reduced 26S levels in both WT and α3ΔN but that the 20S levels only increased in the WT (Fig. 4*B*). This result further indicates a reduced physiological response to paraquat in α3ΔN. Taken together, the resistance of α3ΔN to paraquat is consistent with previous reports that showed oxidative stress resistance when proteasome amounts are increased (55, 56, 57) and adds to the field that intrinsic proteasome activation *via* proteasomal gate opening is also protective against the oxidative stress–inducing toxin, paraquat.

### Resistance to heat shock

Elevated temperatures destabilize protein tertiary structure causing unfolding, protein aggregation, and a cellular heat shock (HS) response. The role of the proteasome in the HS response in multicellular organisms is poorly understood. Although, recent evidence suggests that the 26S proteasome becomes stably activated during HS (87). Nevertheless, given that α3ΔN expression during oxidative stress is protective, we sought to determine whether its expression is also protective under heat-induced stress. The HS was performed by shifting synchronized young adults from 20 °C to 37 °C for 2 h or 2.5 h with a 17 h recovery period followed by survival and paralysis scoring. After the 2 h HS, ∼30% of the WT population was paralyzed while the mutant strain remained largely unaffected (Fig. 4*C*) (*p* < 0.001). When combined with survival data (Fig. 4*D*), 36% of the WT population was adversely affected by a 2 h HS compared to only 2% of α3ΔN (Fig. 4*C*). Since 2 h at 37 °C was not sufficient to cause significant death in either strain (Fig. 4*C*, *p* < 0.05), we increased the HS by 30 min and repeated the analysis. After 2.5 h at 37 °C, α3ΔN paralysis increased slightly to ∼5%, and paralysis of the WT population remained similar to the 2 h exposure (Fig. 4*C*) (*p* = 0.0017). The mortality rate, however, was more significantly impacted; 50% WT population died compared to only 20% of the α3ΔN population (Fig. 4*C*) (*p* < 0.0001). Taken together, the 2.5 h HS adversely impacted 75% of the WT population but only 25% of the mutant population (Fig. 4*D*).

In addition to the phenotypic analysis, we also analyzed the accumulation of polyubiquitinated proteins by Western blot. We quantified the high molecular weight (MW) chain densities after 2 and 2.5 h of HS at 37 °C and normalized each strain to non-HS control. Interestingly, HS increased the high MW chains by 1.95-fold in the WT but only 1.25-fold in the α3ΔN at 2 h. Likewise, the 2.5 h HS induced high MW chains to 1.66-fold in the WT but only 1.30-fold in the α3ΔN (Fig. 4*E*). The two strains are statistically significantly different at 2 h (*p* = 0.027), but these data did not reach significance at the 2.5 h time point (*p* = 0.177), though the trend was consistent (Fig. S5). The differences seen in polyubiquitin chain accumulation suggest the open gate strain may degrade polyubiquitinated proteins more efficiently. This is consistent with observations seen in mammalian cells where expression of α3ΔN in HEK293 cells showed enhanced ubiquitin-dependent degradation of transiently overexpressed Ub-GFP (58). It is also plausible that heat-induced unfolding could allow for ubiquitin-independent degradation by the α3ΔN-20S, preventing the need for polyubiquitination. The ubiquitin blots also showed comparatively more monomeric ubiquitin present in α3ΔN after HS, further indicating a reduced amount of polyubiquitinated species in α3ΔN post HS.

## Conclusion

In this study, we successfully generated a *C. elegans* animal model endogenously expressing a hyperactive, open gate proteasome. Using CRISPR, we generated the open gate proteasome by making an 8 residue deletion from the N-terminal gating region of *pas-3*, which encodes the 20S α3 subunit. The mutation resulted in an open gate proteasome with at least a 13-fold increase in peptide substrate entry and had substantially increased capacity to degrade unstructured and ubiquitinated proteins. Increased degradation of both peptide and protein substrates is consistent with previous studies examining this mutant in yeast and mammalian cells (30, 58). The strain expressing hyperactive proteasomes had a 20% increase in lifespan compared to WT, and the adult nematodes specifically had surprisingly few detrimental phenotypes. The most striking phenotype appeared to be related to embryogenesis causing a substantial reduction in fecundity. Our data also showed that the open gate strain is significantly more resistant to oxidative stress and heat exposure compared to WT. This gate opening mutation also resulted in reduced polyubiquitin accumulation after HS, suggesting that the α3ΔN-26S can degrade ubiquitinated proteins more efficiently than WT or that the α3ΔN-20S is capable of degrading proteins that are misfolded by heat prior to ubiquitination. In addition, our previous studies have also shown that this open channel proteasome mutant is completely resistant to inhibition by some pathological oligomers that can be found in various NDs (49). Future studies currently underway will seek to verify this finding in nematodes in addition to analyzing global proteomic and mRNA expression changes caused by proteasome hyperactivation. Together, the data presented here have shown that expression of a hyperactive, open gate proteasome in a simple multicellular organism is not only feasible but also increases lifespan and resistance to proteotoxic stress. Therefore, these findings support the hypothesis that activating proteasome function *via* gate opening could be a viable and useful approach to increase proteostasis and to potentially treat NDs whereby proteostasis and protein degradation are perturbed.

## Experimental procedures

Detailed materials and methods including proteasome activity assays, *C. elegans* phenotype analyses, and immunoblotting are provided in the supporting information.

## Data availability

The authors declare that data supporting the findings of this study are available within the article and its supplementary information files and are available from the corresponding author upon request.

## Supporting information

This article contains supporting information (43, 49, 88, 89, 90, 91, 92, 93, 94).

## Conflict of interest

The authors declare that they have no conflicts of interest with the contents of this article.
