# Supplementary material for: Hyperactivation of the proteasome in Caenorhabditis elegans protects against proteotoxic stress and extends lifespan
Source: J Biol Chem. 2022 Aug 23;298(10):102415. doi: 10.1016/j.jbc.2022.102415 (PMC9486566; doi:10.1016/j.jbc.2022.102415)
Supplement: Supporting information and Figures S1–S5 [file mmc1.pdf]

Supporting information for:

Hyperactivation of the proteasome in *Caenorhabditis elegans* protects against proteotoxic stress and extends lifespan

Raymond T. Anderson, Thomas A. Bradley, David M. Smith*

Department of Biochemistry, West Virginia University School of Medicine, 64 Medical Center Dr., Morgantown, WV 26506

* David M. Smith

Email: dmsmith@hsc.wvu.edu

Keywords: *Caenorhabditis elegans* (*C. elegans*), enzyme kinetics, Oxidative stress, proteotoxic stress, proteasome, protein degradation, ubiquitin, toxicity, aging

This file contains:

Materials and Methods

Supporting Figures and Figure legends 1-4

Materials and Methods

Strains and Maintenance. Standard methods of culturing *C. elegans* were followed(1). All strains were cultured at 20˚C on standard NGM plates seeded with OP50. Wild-type and CRISPR edited strains used in this study were obtained from InVivo Biosystems (Oregon, USA). All strains used include: N2, COP1857 *pas-3*(*knu*736 [NTD del]), and COP1858 *pas-3*(*knu*737 [NTD del]). Most experiments were performed with both mutant clones and yielded similar results. Data shown are primarily from COP1857 *pas-3*(*knu*736 [NTD del]). Age synchronization was performed through alkaline bleaching discussed previously(2). Well-fed gravid adults from 2 10cm plates were collected and washed 3 times with ddH$_2$O in 15mL conical tubes. Water was aspirated to 3.5mL and 1.5mL freshly prepared bleach/NaOH mixture was added [1mL 5% sodium hypochlorite (Fisher Scientific) and 0.5mL 5N NaOH. The nematode/bleach solution was vortexed every 2 minutes for 10 minutes until no nematode fragments remained. Sterile M9 was added to the solution to neutralize the reaction and the tubes were centrifuged at 3,000xg for 1 minute to pellet the eggs. Eggs were washed with 10mL sterile M9, centrifuged again for at 3,000xg for 1 minute, and M9 was discarded. The pelleted eggs were added to fresh 15mL conical tubes containing 7.5mL sterile M9 and incubated overnight with light agitation to allow for hatching. The resulting L1 arrested nematodes were harvested within 24hrs, plated on *E. coli* (OP50) seeded plates, and incubated in a 20˚C incubator until desired stage.

CRISPR-Cas9 approach. The CRISPR/Cas9 system was used with homology-directed repair (HDR) as described previously(3) to precisely delete amino acids 2-9 of *pas-7*. The strains produced are COP1857 *pas-3*(*knu*736 [NTD del]), and COP1858 *pas-3*(*knu*737 [NTD del]). The strains were made and provided by InVivo Biosystems (formerly NemaMetrix). Co-CRISPR: The *dpy-10* co-CRISPR method described previously (4) was used to identify animals in which the CRISPR/Cas9 system was active. The injection strain used in DSMI01 was N2, obtained from the *C. elegans* Genetics Center (CGC) (University of Minnesota, Minneapolis). Single guide RNA sequences used include:

5' sgRNA sequence: 5' - TCGTTCTACTATCGTAACGA - 3'

3' sgRNA sequence: 5' - AACGACCATCTTCTCTCCGG - 3'

DNA extraction for PCR and off-target effects analysis. Genomic DNA (gDNA) was extracted for whole genome sequencing (WGS) and genotyping via PCR. For WGS, two 10cm plates of *pas-3*(knu746 [NTD del]) and *pas-3*(knu747 [NTD del]) were collected and washed several times with M9 to remove residual bacteria. Washed populations were resuspended in lysis buffer [1x High Fidelity (HF) buffer (New England Biolabs) + 1mg/mL Proteinase K (Thermo Scientific)], frozen at -80˚C for 15 minutes, heated to 65˚C for 1hr for lysis and 90˚C for 20˚C to deactivated proteinase K. Cellular debris were spun down at 16,000xg and the supernatant containing gDNA was collected and sent to Psomagen (Rockville, Maryland, USA) for sequencing and off target effect analysis against WT (Bristol N2) reference genome (WBcel235). For PCR genotyping, a single nematode was lysed in 10µL lysis buffer as described above. PCR was performed in 10µl PCR buffer [OneTaq (NEB), 1µL gDNA, and .5µM of Forward and Reverse Primers] with standard temperature parameters (see below). Products were separated on 2% agarose gel containing ethidium bromide and imaged using Gel Doc XR imaging System (Bio-Rad). WT PCR amplifications run at 331bp, while mutant PCR bands run at 317bp. Primers and thermocycler conditions listed below.

Primers used:

- WT forward: gaATGACTATTTTTAGTCCGGAGGGA
- α3ΔN forward: TCGTTACGATAGTAGAACGACCATC
- Reverse (used for both strains): ATTCTGGACGAGCTGCTCAACT

Thermocycler conditions:
- Initial Denaturation: 98˚C
- Cycles (35x): 98˚C, 50˚C, 72˚C
- Final extension: 72˚C
- Hold: 4˚C

Protein extraction. Protein lysates were extracted by collecting two 10cm plates of synchronized young adults and washing several times with M9. The clean nematode pellets were then resuspended in appropriate ice-cold lysis buffer depending on the application: 20S lysis buffer: [50mM Tris-HCl pH.7.4, 1mM DTT, 5% Glycerol], 26S lysis buffer: [50mM Tris-HCl pH 7.4, 1mM DTT, 10% glycerol, 2mM ATP, 5mM $MgCl_2$]. Samples were then sonicated using Sonic Dismembrator Model 100 (Fisher Scientific) at an output power of 1 (5 x 10 pulses) until no nematode fragments were visible. Samples were then spun down at 16,000xg for 15min to pellet cellular debris. The supernatant was collected, and protein concentration was determined using Coomassie Plus (Bradford) Protein Assay (Thermo Scientific). Samples were either used immediately or stored at -80˚C for future use.

Proteasome activity assays – fluorogenic substrates. 20S and 26S Proteasome activity in *C. elegans* lysates (5µg) was measured using fluorogenic peptides or protein substrates as described(5, 6) in 96-half-well non-binding surface treated black plates (corning) at 20˚C. To measure 20S activity, lysates were added to a reaction buffer containing 50 mM Tris–HCl (pH 7.4), 2mM DTT, and 100 µM fluorogenic substrate (Suc-LLVY-AMC, ac-LLE-AMC, Boc-LRR-AMC). To measure 26S activity, lysates were added to reaction buffer appropriate for ATP hydrolysis [50mM Tris-HCl (pH 7.4), 100µM Suc-LLVY-AMC, 2mM DTT, 2mM ATP, and 5mM $MgCl_2$]. Fluorescence was measured every 60S for 2hrs (ex/em: 380/460 nm). Activity was calculated as the rate of increase in fluorescence intensity over time (rfu/min) ± SD (N=3). Experiments were repeated at least twice.

Proteasome activity assay – protein substrates. Unstructured protein degradation was measured in 50µl reactions using 96-half-well non-binding surface treated black plates (Corning) at 20°C. *C. elegans* lysates (5µg) were incubated in reaction buffer with ATP/$MgCl_2$[50mM Tris-HCl pH 7.4, 1mM DTT, 5% glycerol, 2mM ATP, 5mM $MgCl_2$] or without ATP/$MgCl_2$ [50mM Tris-HCl pH 7.4, 1mM DTT, 5% glycerol] containing 0.8µg FITC-casein (Sigma). Fluorescence was measured every 2min for 4hrs using the Synergy 2 Microplate reader (BioTek). Fluorescence polarization (FP) was calculated in Gen5 software version 2.0. Degradation rates were determined by calculating the total change in FP over 4 hours. Ubiquitin dependent degradation was assessed using a recombinantly expressed linear tetraubiquitin fused to a circularly permuted GFP with a 35-residue unstructured region on its N-terminus (Ub_4(lin)-cpGFP-35) (gift from Andreas Matouschek) as described (6). Degradation rate was calculated by determining the half-life ($t_{1/2}$) of GFP fluorescence using one phase decay fit in GraphPad (Prism) (N=3). Experiments were performed at least twice.

In-gel proteasome activity assay and native western blot. Native-PAGE in-gel 20S/26S proteasome activity assay was performed as described previously(6). Protein lysates (20µg) were mixed with Novex™ Tris-glycine Native Sample Buffer (Invitrogen) and separated on NuPAGE™ 3-8% Tris Acetate gels (Invitrogen) using Novex™ Tris-glycine Native Running Buffer (Invitrogen) containing 1mM DTT, 2mM ATP, and 5mM $MgCl_2$ at 4˚C and 50V overnight. The gels were then incubated with slight agitation in 26S activity buffer [50mM Tris-HCl pH 7.4, 2mM DTT, 5% glycerol, 2mM ATP, 5mM $MgCl_2$, and 100µm Suc-LLVY-AMC] for 30min at 20˚C and visualized using ethidium bromide imaging protocol on G:BOX XX9 (Syngene). Gate-opening was induced by incubating in activity buffer + 0.02% SDS for 30min at 20˚C and imaged as described above. To perform immunoblot, the gel was transferred to Immobilon®-FL PVDF membrane at 30V overnight. The membrane was blocked in tris buffered saline + 0.01% tween20 (TBST) +10% nonfat milk for 1hr at room temperature (~20˚C), briefly washed and incubated with primary antibody (Anti-Proteasome α's1,2,3,5,6,7 subunits, Enzo, MCP231; Anti-PSMD7/Mov34, ab140428) diluted 1:1000 in TBST+5% nonfat milk at 4˚C overnight, washed (3 x 5min), washed with TBST (3 x 5min), incubated with secondary antibody (DyLight™ 550, Thermo, 10173; Alexa Fluor Plus 680, A32729) diluted 1:3000 in TBST+ 5% nonfat milk for 1hr at room temperature, washed with TBST (3 x 5 minutes), and imaged using Amersham Typhoon (GE). The image shown is representative of 3 biological replicates. Note regarding native western: We repeatedly saw less 20S signal in the 26S band than typically expected. We speculate that in these native western conditions may allow for some steric blocking of the 20S epitope in the *c. elegans* 26S complex (e.g. Fig 2C), though other explanations are also possible.

Western blot – proteasome. The antibody used to determine proteasome levels were purchased through Enzo (Anti-Proteasome α's1,2,3,5,6,7; MCP231)). The antibody used to label the loading control, tubulin, was purchased through abcam (anti-Tubulin YOL1/34, ab6161).". The primary antibodies were diluted 1:1000 in TBST + 3% bovine serum albumin (BSA). Secondary antibodies were purchased through Invitrogen (DyLight™ 800, 10024; Alexa Fluor™ Plus 680, A32729) and diluted 1:3000. 100µg of lysate from each strain was separated on NuPAGE™ Bis-Tris 4-12% (Invitrogen) gel and transferred to Immobilon-FL PVDF membranes (Millipore). Membranes were blocked for 1hr at room temperature in TBST+3% BSA, briefly rinsed with TBST, incubated with primary antibodies overnight at 4˚C, washed with TBST (3 x 5min), Incubated with secondary antibodies for 1hr at room temperature, and washed with TBST (3 x 5min), and imaged using Amersham Typhoon (GE). Signal intensities were quantified using ImageJ and statistical significance was determined using an unpaired Student's t-test (N=3) (Excel).

Lifespan. Synchronized day one adults were collected and used to perform lifespan analysis as described(7). Young adult nematodes were collected and divided between 6 OP50 seeded NGM plates containing floxuridine (FUdR, RPI) to halt reproduction (100 µg/mL). Individuals were counted every 1-2 days and scored as dead if there was no movement after mechanical stimulation. Nematodes were scored as "censored" if their death was caused by unnatural events such as drying out, internal hatching, or vulvar protrusion. Nematodes were transferred to new plates every 2-3 days to avoid starvation. Kaplan-Meyer curves were generated followed by log-rank analysis using GraphPad Prism version 9.0.0 (San Diego, California USA)(N≥120). A value of $P<0.05$ was considered statistically significant. All experiments were performed at 20˚C and at least 3 times under blinding conditions.

Length measurements. Stemi 508 (ZEISS) stereomicroscope and Axiocam 208 color (ZEISS) were used at 10X magnification to capture random images of each strain at various stages of development. The software ZEN Digital Imaging for Light Microscopy (RRID:SCR_013672) was used to take manual measurements of individual nematodes. Data shown represents the average length of each strain during each stage of development ($N \geq 3$) ± SD. Statistical significance was calculated using unpaired Student's t-test in GraphPad (Prism 9). A value of $P<0.05$ was considered statistically significant.

Developmental Timeline. Developmental timeline was examined using the wMicroTracker™ (WMT) from InVivo Biosystems and the experimental design was adapted from a previous study(8). Synchronized L1 arrested nematodes from each strain were added to 95µL S-complete supplemented with OP50 *E. coli* (OD_{600}:0.07) in 8 wells of a clear U-bottom 96-well plate (Corning) with about 100 nematodes per well. The plate was placed inside the WMT, and activity was monitored for 72hrs at room temperature (~20˚C). The mean activity in each 30min time interval of 8 technical replicates for each strain was generated with the WMT software and plotted using GraphPad (Prism). The data shown is the 2nd order smoothing of the raw activity counts over 72hrs (N=8).

Food Consumption. Food consumption was measured as described previously(9) using liquid S-complete medium with 100µg/mL Streptomycin and OP50 (OD_{600}: 1-1.5) in black, optically clear flat-bottom 96-well plates (Corning). 20-30 Age synchronized L1 larvae were washed and placed into each well with 12 technical replicates per strain and the OD_{600} was measured using the synergy MX plate reader (Biotek). The plates were then covered with a gas permeable seal (BrandTech® Scientific, Inc.) and incubated at 20˚C. Every 24hrs, plates were shaken at 1,200rpms for 25 minutes to resuspend the bacteria; the OD_{600} was measured, and the plate was resealed and returned to 20˚C. On day 4 of adulthood, the number of nematodes in each well were counted to normalize changes in OD_{600}. Daily food consumption was determined by subtracting the OD_{600} of the test well from the average OD_{600} of wells containing no nematodes (control), divided by the number of individuals in the test well, and subtracted from the absorption of that well the previous day. The data is represented as the average daily consumption each day (N=12) ±SD. Statistical significance was calculated using an unpaired Student's t-test in GraphPad (Prism). A value of $P<0.05$ was considered statistically significant.

Pharyngeal pumping. Pharyngeal pumping was manually counted using Stemi 508 (ZEISS) at 50X magnification in 10s intervals to determine pumps per minute. Statistical significance for each day was calculated using an unpaired Student's *t*-test in GraphPad (Prism) and a value of $P<0.05$ was considered statistically significant (N=15).

Paraquat treatment. Paraquat was used to measure oxidative stress sensitivity as described previously(10). 20 synchronized L4 or Day 1 adult nematodes from each strain were placed on 5 NGM plates containing 100mM paraquat and seeded with OP50. Each individual nematode was counted every hour for 25hrs and scored as dead or censored. Each Plate was counted as a replicate within each strain and statistical differences at each timepoint was calculated using an unpaired t-test (Prism 9). Experiments were performed twice under blinding conditions. Values shown are mean survival ± SEM, (N=5). A Value of $P<0.05$ was considered statistically significant.

Heat-shock treatment. Heat-shock (HS) experiments were designed based on the methodological considerations discussed previously(11). For each strain, 120 age-

synchronized, young-adult worms were divided between 6 OP50 seeded NGM plates (20 worms/plate) at room temperature for each condition. Plates were placed in a 37°C incubator for either 2hrs or 2.5hrs then returned to 20°C for a 17hr recovery period. After recovery, the individual nematodes were counted and scored as dead, paralyzed, or censored. Death was characterized by absolutely no movement following mechanical stimuli, and paralysis was characterized by the inability to move more than half its body length following mechanical stimuli. Bar graphs represent the average amount of death or paralysis of 6 plates (20 nematodes each) ±SD (N=6). Unpaired Student's t-tests were used to identify significant differences between strains. A value of $P<0.05$ was considered statistically significant. The 2hr and 2.5hr conditions were performed twice with separate populations, and experiments were performed under blinding conditions.

Western blot – Ubiquitin. Primary antibodies used to detect ubiquitin were purchased through Abcam and ABclonal (anti-ubiquitin Ubi-1, ab7254; anti-βActin, AC026) and diluted 1:1000 in TBST+3% BSA. The secondary antibodies used include IgG-HRP (Thermo, 32430) diluted 1:10,000 in TBST+3%BSA and Alexa Fluor™ 488 (Thermo, A-11008) diluted 1:3000 in TBST+3%BSA. To probe for HS induced ubiquitin accumulation, young adult nematodes were incubated at 37˚C for 2hrs and 2.5hrs. 1hr after HS, 100 individual nematodes were transferred to 30µL M9 and placed at -80˚C for at least 15 minutes. Samples were then thawed, mixed with 10µL LDS Sample Buffer (4X) (Invitrogen), and lysed by incubating at 95˚C for 10min. Lysed samples were separated using NuPAGE® 4-12% Bis-Tris gels (Invitrogen) and transferred to Immobilon-FL PVDF membranes (Millipore) using NuPAGE® Transfer Buffer (Invitrogen) at room temperature (30V, 90min). Membranes were blocked for 1hr at room temperature in TBST+3% BSA, briefly rinsed with TBST, incubated with primary antibodies overnight at 4˚C, washed with TBST (3 x 5min), incubated with secondary antibodies for 1hr at room temperature, and washed with TBST (3 x 5min). HRP-labeled ubiquitin was detected using Pierce ECL-Plus Substrate (Thermo) and imaged using G:BOX XX9 (Syngene). Fluorescent antibodies were imaged using Amersham Typhoon (GE). Intensities and ratios were calculated using ImageJ (NIH).Fold changes were calculated by determining the ratio between the HS conditions and corresponding control for each strain. The data were analyzed using 2-way ANOVA in GraphPad (Prism, N=2) A value of $P \leq 0.05$ was considered statistically significant.

Statistical analysis. The data were analyzed using unpaired Student's t-tests, 2-way ANOVA, and log-rank tests (Prism). N-values are provided in each method. For all statistical analyses, a value of $P<0.05$ was considered significant (*$P \leq 0.05$, **$P \leq 0.01$, ***$P \leq 0.001$, ****$P \leq 0.0001$).

References

1. Stiernagle, T. (2006) Maintenance of *C. elegans. WormBook*. 10.1895/wormbook.1.101.1
2. Porta-de-la-Riva, M., Fontrodona, L., Villanueva, A., and Cerón, J. (2012) Basic *Caenorhabditis elegans* methods: Synchronization and observation. *J. Vis. Exp.* 10.3791/4019
3. Paix, A., Folkmann, A., Rasoloson, D., and Seydoux, G. (2015) High efficiency, homology-directed genome editing in *Caenorhabditis elegans* using CRISPR-Cas9 ribonucleoprotein complexes. *Genetics*. **201**, 47–54
4. Arribere, J. A., Bell, R. T., Fu, B. X. H., Artiles, K. L., Hartman, P. S., and Fire, A. Z. (2014) Efficient marker-free recovery of custom genetic modifications with CRISPR/Cas9 in *Caenorhabditis elegans*. *Genetics*. **198**, 837–846
5. Smith, D. M., Chang, S. C., Park, S., Finley, D., Cheng, Y., and Goldberg, A. L. (2007) Docking of the proteasomal ATPases' carboxyl termini in the 20S proteasome's α ring opens the gate for substrate entry. *Mol. Cell*. **27**, 731–744
6. Thibaudeau, T. A., Anderson, R. T., and Smith, D. M. (2018) A common mechanism of proteasome impairment by neurodegenerative disease-associated oligomers. *Nat. Commun.* 10.1038/s41467-018-03509-0
7. Sutphin, G. L., and Kaeberlein, M. (2009) Measuring *Caenorhabditis elegans* life span on solid media. *J. Vis. Exp.* 10.3791/1152
8. Hunt, P. R., Olejnik, N., Bailey, K. D., Vaught, C. A., and Sprando, R. L. (2018) *C. elegans* Development and Activity Test detects mammalian developmental neurotoxins. *Food Chem. Toxicol.* **121**, 583–592
9. Gomez-Amaro, R. L., Valentine, E. R., Carretero, M., Leboeuf, S. E., Rangaraju, S., Broaddus, C. D., Solis, G. M., Williamson, J. R., and Petrascheck, M. (2015) Measuring food intake and nutrient absorption in *Caenorhabditis elegans*. *Genetics*. **200**, 443–454
10. Senchuk, M., Dues, D., and Van Raamsdonk, J. (2017) Measuring oxidative stress in *Caenorhabditis elegans*: Paraquat and juglone sensitivity assays. *BIO-PROTOCOL*. 10.21769/bioprotoc.2086
11. Zevian, S. C., and Yanowitz, J. L. (2014) Methodological considerations for heat shock of the nematode *Caenorhabditis elegans*. *Methods*. 10.1016/j.ymeth.2014.04.015

A. B.

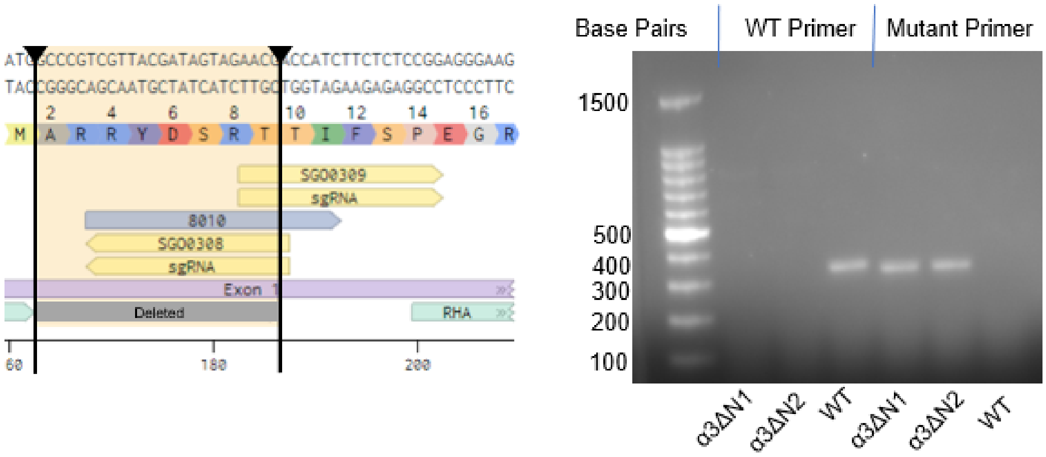

C.

- On-target region

Chromosome	Position	Sequence	Direction	Mismatches	Variants in target region ± 20bp
chrI (NC_003279.8)	5105999	AACGACCATCTTCTCTCCGGAGG	-	0	Deletion
chrI (NC_003279.8)	5106017	TCGTTCTACTATCGTAACGACGG	+	0	Deletion

- Off-target region (mismatch ≤ 4)

Chromosome	Position	Sequence	Direction	Mismatches	Variants in target region ± 20bp
chrIV (NC_003282.8)	10374594	TCGTTtcACTATCGgAACaACGG	-	4	-
chrV (NC_003283.11)	5608759	AACGACCATCcTaTtTCCGaAGG	+	4	-
chrV (NC_003283.11)	16855932	AACGACaATCTatTCTCCGcCGG	+	4	-
chrII (NC_003280.10)	9485001	AACGcCCATCTTtctTCCGGAGG	+	4	-
chrX (NC_003284.9)	12879032	AACGAaCATCTTagCTtCGGTGG	-	4	-

Fig. S1. Molecular approach to gene editing of the *C. elegans* DNA using Crispr/Cas9 and its verifications

A. CRISPR approach showing guide RNA in yellow and region deleted in gray.

B. PCR genotyping showing amplification to each primer set. The CRISPR edited nematodes each have a 317bp mutant band and do not have the 331bp wild type band and vice-versa.

C. On-target and off-target editing analysis showing deletion in targeted region and no variations in off-target regions. Off-target regions were detected by finding regions that have a complimentary nucleotide sequence with ≤4 mismatches to the guide RNA used to perform the gene editing in the target region. The dashed line means no variations within 20bp of the off-target region identified.

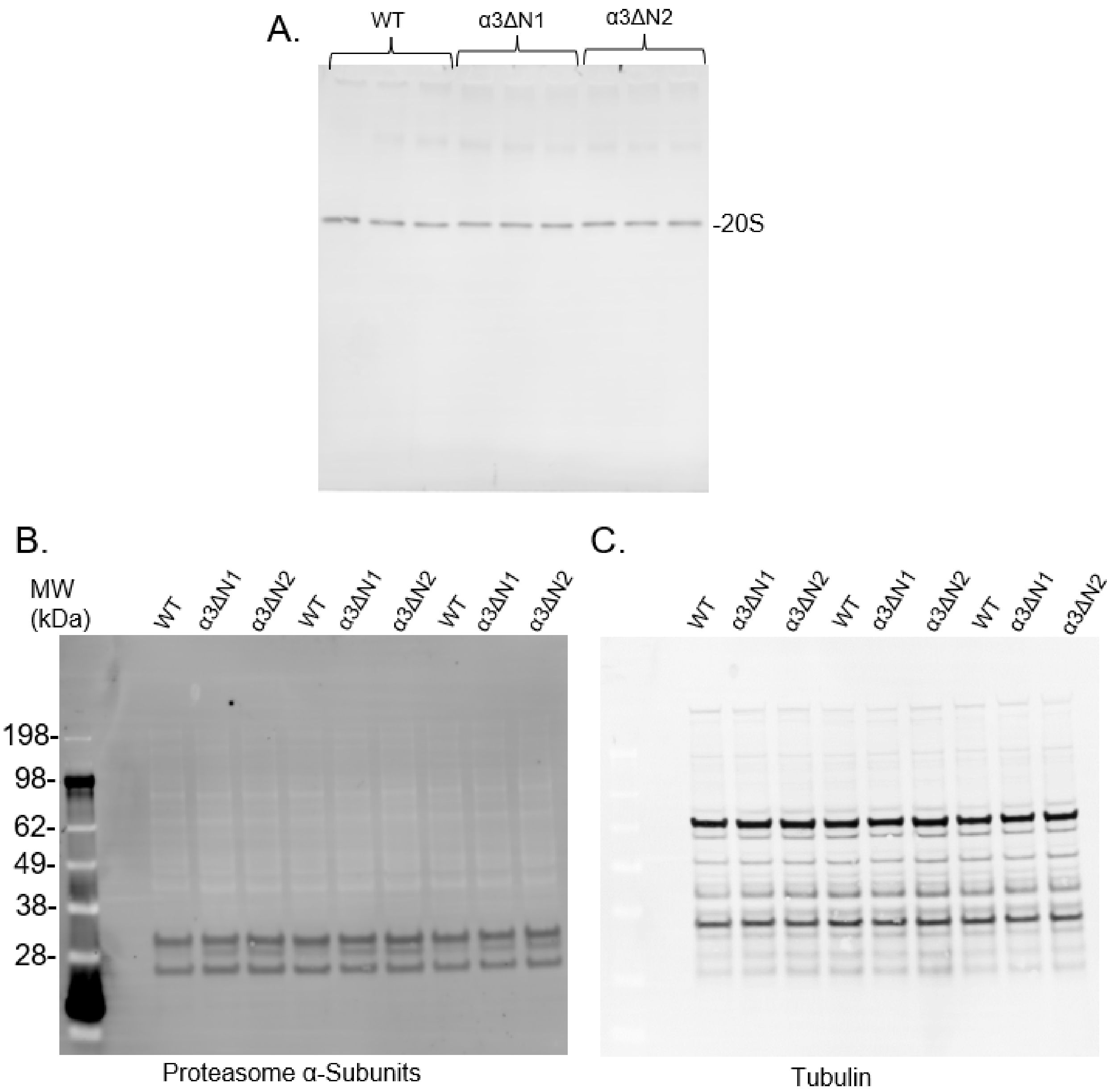

Figure S2. Native-PAGE western blot and full SDS-PAGE immunoblots shown in figure 1C.

A. Native-PAGE western blot probing for 20S α-subunits (Enzo, MCP231).

B. 20S α-subunits **corresponding to Fig. 1C** (Enzo, MCP231).

C. Tubulin bands used for loading control **corresponding to Fig. 1 C** (abcam, ab6161).

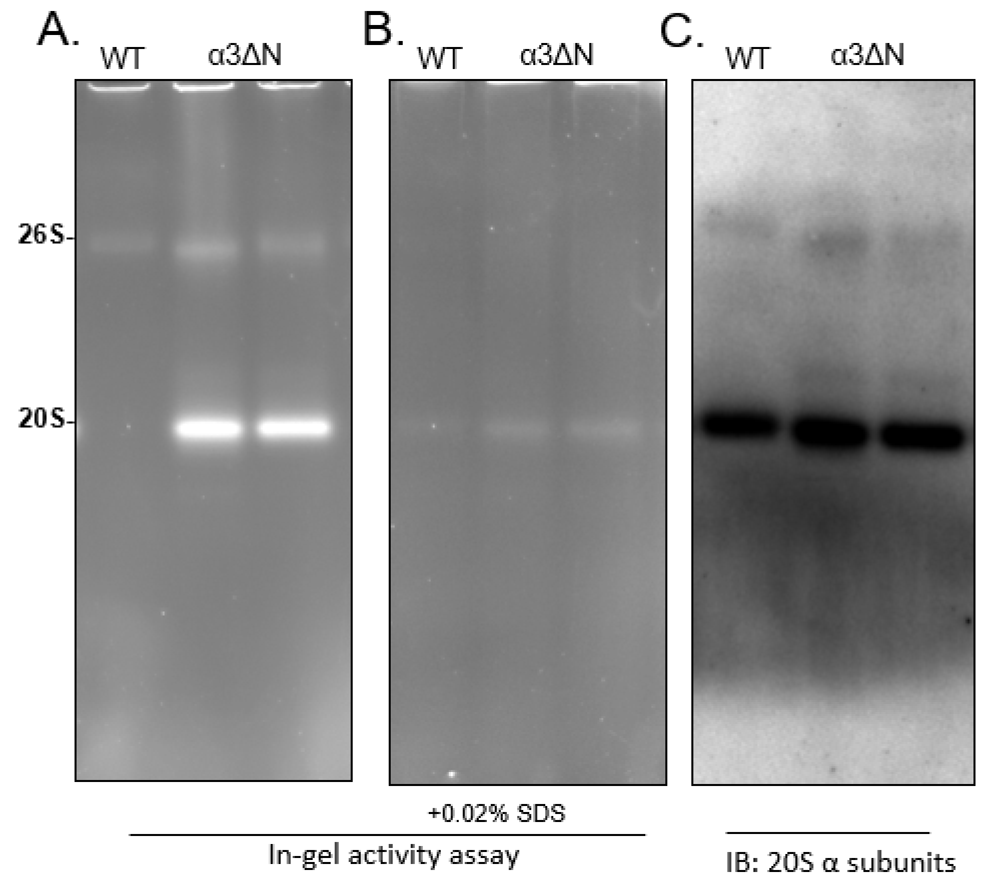

Fig. S3. Full In-gel activity assay and western blot **corresponding to Fig. 2C**.

A. In-gel activity assay showing 20S and 26S Suc-LLVY-AMC peptidase activity after 30 minutes.

B. In-gel activity assay after incubation with 0.02% SDS for 30 minutes.

C. Native-PAGE immunoblot of 20S α-subunits (Enzo, MCP231).

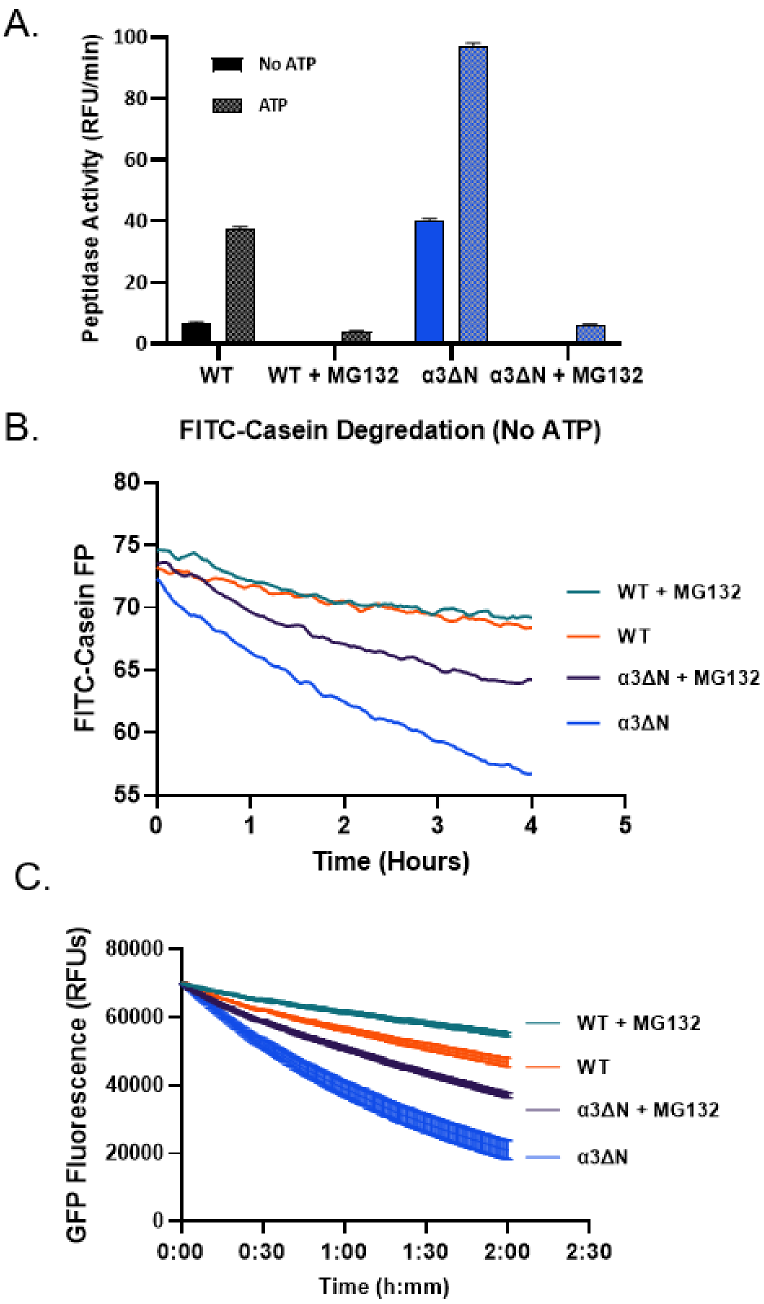

Fig. S4. In-vitro proteasome activity assay without normalization to MG132 corresponding to Figure 2.

A. Suc-LLVY-AMC peptidase activity with and without ATP ± MG132 (100μM). Values shown are the mean ± SD (N=3)

B. Fluorescence polarization (FP) of FITC-Casein over 4hrs using 5ug lysate from each strain without ATP (100μM MG132 used to inhibit proteasome activity). Values are the 2nd order smoothing of the raw FP at each timepoint (N=3).

C. GFP fluorescence over time showing Ub_4(lin)GFP-35 degradation ± MG132. Error bars represent SD (N=3)

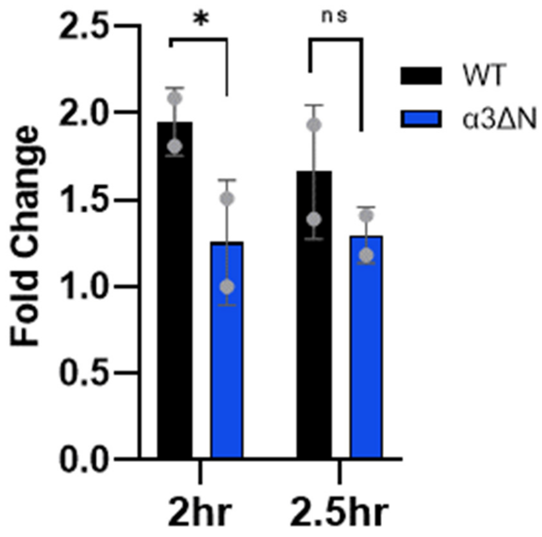

Fig. S5. Ubiquitin accumulation after HS quantification corresponding to Figure 4E. The high molecular weight ubiquitin (anti-ubiquitin Ubi-1, ab7254) signal was calculated from 2 separate experiments and normalized to the loading control, actin (anti-βActin, AC026). HS induced high molecular weight accumulation in each strain was normalized to the corresponding non-HS control (first 2 lanes) to calculate fold change (FC). Statistics were performed using a 2-way ANOVA. Bar graph shows average FC ± SD (N=2); *P≤0.05.
